# Case Report: Treatment of Anti-MDA5-Positive Amyopathic Dermatomyositis Accompanied by a Rapidly Progressive Interstitial Lung Diseases With Methylprednisolone Pulse Therapy Combined With Cyclosporine A and Hydroxychloroquine

**DOI:** 10.3389/fmed.2020.610554

**Published:** 2020-11-27

**Authors:** Qia-Chun Zhang, Min-Ying Liu, Zhi-Xin Chen, Yimin Talia Chen, Chang-Song Lin, Qiang Xu

**Affiliations:** ^1^Guangzhou University of Chinese Medicine, Guangzhou, China; ^2^Bao'an Traditional Chinese Medicine Hospital Affiliated to Guangzhou University of Chinese Medicine, Shenzhen, China; ^3^Department of Rheumatology, The First Affiliated Hospital of Guangzhou University of Chinese Medicine, Guangzhou, China; ^4^The Chinese Medicine Department, South China Agricultural University Hospital, Guangzhou, China; ^5^University of Rochester, Rochester, NY, United States

**Keywords:** combination treatment strategy, interstitial lung diseases, anti-MDA antibody, cyclosporine A, hydroxychloroquine, methylprednisolone pulse therapy, amyopathic dermatomyositis

## Abstract

**Introduction:** Patients with anti-melanoma differentiation-associated gene 5 (MDA5) antibody-positive amyopathic dermatomyositis (ADM) often develop rapidly progressive interstitial lung diseases (RP-ILD), with poor treatment success. Many studies have shown that this is the main cause of death in patients with anti-MDA5 antibody-positive ADM.

**Case Presentation:** A 37-years-old woman developed a cough, shortness of breath, and a rash on both hands, which resembled Gottron's signs. Upon laboratory examination, the results were as follows: antinuclear antibody (ANA) positive; anti-Ro52 antibody positive; and anti-MDA5 antibody positive. Pulmonary high-resolution CT (HRCT) scan showed pulmonary interstitial inflammatory changes, and mediastinal and subcutaneous emphysema. She was finally diagnosed with anti-MDA5 antibody-positive ADM accompanied by RP-ILD. She was first given high-dose-steroid pulse therapy with methylprednisolone (500 mg per day for 3 days) followed by methylprednisolone (40 mg, daily), cyclosporine A (100 mg, twice per day), and hydroxychloroquine (200 mg, twice per day). Since her discharge from our hospital in March of 2018, she has maintained the methylprednisolone therapy (tapered to 10 mg daily), cyclosporine A (100 mg, twice per day), and hydroxychloroquine (200 mg, twice per day).

**Outcomes:** Pulmonary HRCT scans taken on 4, 9, and 26 months after her discharge from our hospital showed that the interstitial pneumonitis had significantly improved and that mediastinal and subcutaneous emphysema had been gradually absorbed. The patient can now participate in regular work and activities of daily living.

**Conclusion:** The treatment of methylprednisolone pulse therapy combined with cyclosporine A and hydroxychloroquine may be an option for the RP-ILD accompanied by anti-MDA-positive ADM. After the acute phase, this combination therapy strategy is helpful to the disease control of patients.

## Introduction

Idiopathic inflammatory myopathies (IIMs) are heterogeneous diseases characterized by symmetrical proximal muscle weakness, elevated muscle enzymes, and chronic skin or muscle inflammation (1). Since the 1970s, several sets of standards have been published for the classification and/or diagnosis of IIMs (2). In 1975, Bohan and Peter proposed several subgroups of IIMs: polymyositis (PM), dermatomyositis (DM), juvenile dermatomyositis, overlap myositis, and myositis associated with cancer (3, 4). In 2005, Troyanov et al. proposed a classification system based on clinical–serological definitions and introduced a new subgroup that was called clinicoserologic overlap myositis (5). Currently, IIM is most often classified into PM, DM, and inclusion body myositis (1). Amyopathic dermatomyositis (ADM) is a clinical subtype of DM, distinguished from other DM subtypes by presentation without symptoms of muscular disease. Euwer et al. first reported six cases of DM without evidence of muscle disease in 1991 (6). Sontheimer formally defined ADM in 2002 (7). According to the Classification and Diagnostic Criteria for IIMs released by the European Center for Neuromuscular Diseases and the American Myopathy Research Collaborative Group in 2004, ADM patients have typical rash manifestations of DM. ADM patients do not have objective muscle weakness, and their creatine kinase (CK) levels and electromyograms are normal.

ADM accompanied by rapidly progressive interstitial lung diseases (RP-ILD) has been reported mainly in Asia, with low treatment success (8). To our knowledge, there is no clinical trial for this disease, since the prevalence is too low and only a few cases are reported. The diagnosis of interstitial lung diseases (ILDs) is based on abnormal imaging findings with respiratory symptoms (9). Patients with ILDs often present with active dyspnea, restrictive ventilation disorder, decreased diffusion function, and hypoxemia. Pathologically, ILD is characterized by diffuse pulmonary parenchyma, alveolar inflammation, interstitial fibrosis, and diffuse shadow on chest X-rays. According to the clinical manifestations of ILDs, patients were divided into two types: acute/subacute type and chronic type (9). According to the International Consensus Statement on Idiopathic Pulmonary Fibrosis of the American Thoracic Society and the European Respiratory Society, RP-ILD is defined as a progressive ILD within 3 months after the onset of respiratory symptoms (10). Suda et al. reported that acute/subacute ILDs were generally resistant to drugs, while chronic ILDs responded well. At the same time, the mortality rate of acute/subacute ILDs was much higher than that of chronic ILDs (67 and 0%, respectively) (9).

There may be a link between ADM and ILDs, as postulated by Nakashima et al. in their study of DM specific autoantigens, and melanoma differentiation-associated gene 5 (MDA5) is a serological marker of both DM and ILDs (9). The 6-months and 5-years mortality rates of patients with anti-MDA5 antibody-positive ADM were significantly higher than those of patients with anti-MDA5 antibody-negative ADM (11). Patients with anti-MDA5 antibody-positive ADM often develop RP-ILDs with poor prognosis. Koga et al. reported that the death of all anti-MDA5 antibody-positive patients was attributed to RP-ILD respiratory failure (11). The research of Gono et al. pointed out that the cumulative 100-months survival rate was 66% for the entire anti-MDA-positive ADM patient group, and the cumulative 100-months survival rates were significantly lower in the RP-ILDs subset than in the non-RP-ILDs subset (log-rank test, *P* = 0.039). Fatal outcomes occurred remarkably often within the first 6 months (12). Anti-MDA5 antibody-positive ADM accompanied by RP-ILDs is generally treated via pharmacological methods. High-dose corticosteroids and immunosuppressants are commonly used treatments (13–16), but most of them have poor efficacy and prognosis.

In this study, we reported a case of anti-MDA5 antibody-positive ADM accompanied by RP-ILDs and performed a potential treatment that methylprednisolone pulse therapy combined with cyclosporine A and hydroxychloroquine.

## Case Presentation

On October 2017, a 37-years-old woman developed polyarticular pain and swelling in her limbs, accompanied by morning stiffness and fever. The morning stiffness lasted a few minutes, and the highest body temperature was 39°C. At the onset of her fever, the skin around the joint was warm to touch. Upon a visit to another hospital, the details of the test results are as shown in Table 1 (adult reference values in parentheses). She was tentatively diagnosed with connective tissue disease (CTD) and treated with prednisone (10 mg per day), hydroxychloroquine (200 mg per day), and loxoprofen sodium (60 mg per day).

**Table 1:** Laboratory test results.

	**5 months before baseline**	**4 months before baseline**	**Baseline**
**PERIPHERAL BLOOD**			
WBC (4.0–10.0 × 10e9/L)		7.8 × 10e9/L	10.21 × 10e9/L
NEU (2.0–7.5 × 10e9/L)		5.23 × 10e9/L	8.36 × 10e9/L
LYM (1.6–4.0 × 10e9/L)		1.51 × 10e9/L	0.94 × 10e9/L
**IMMUNOLOGICAL TESTS**			
Anti-CCP antibody	Negative	Negative	
ANA	Positive	Positive	Positive
Anti-dsDNA antibody	Negative		Negative
Anti-Ro52 antibody		Positive	Positive
Anti-MDA5 antibody			Positive
**BLOOD GAS ANALYSIS**			
PO_2_ (83.0–108.0 mmHg)			63.5 mmHg
SaO_2_ (95.0–99.0%)			91.4%
O_2_Hb (94.0–98.0%)			90.3%
**BLOOD CHEMISTRY**			
CK		Normal	Normal
CK-MB		Normal	Normal
DDi (0–0.55 mg/L)			2.26 mg/L
LDH (120–250 U/L)			441 U/L
α-HBDH (72–182 U/L)			345 U/L
**SEROLOGICAL TESTS**			
ESR (0–20 mm/h)	43 mm/h	38 mm/h	36 mm/h
CRP (0–8 mg/L)	Negative	11.4 mg/L	24.1 mg/L
IgG (7.51–15.6g/L)			16.9 g/L

*WBC, Leukocyte Count; NEU, Neutrophil count; LYM, Lymphocyte count; anti-CCP antibody, anti-cyclic citrullinated peptide antibody; ANA, antinuclear antibody; anti-ds-DNA antibody, anti-double-stranded DNA antibody; MDA5, melanoma differentiation-associated gene 5; PO_2_, partial pressure of oxygen; SaO_2_, Oxygen saturation; O_2_Hb, Oxygenated hemoglobin fraction; CK, creatine kinase; CK-MB, Creatine Kinase, MB Form; DDi, D-dimer; LDH, lactate dehydrogenase; α-HBDH, α-hydroxybutyrate dehydrogenase; ESR, Erythrocyte sedimentation rate; CRP, C-reactive protein; IgG, immunoglobulin G*.

After 2 weeks, the fever subsided. The polyarticular pain, swelling, and morning stiffness in her limbs improved but did not fully resolve. The patient subsequently developed a cough, shortness of breath, and Raynaud's phenomenon and was admitted to our hospital on November 2017. Our tests are shown in Table 1 (adult reference values in parentheses). Pulmonary high-resolution CT (HRCT) scan showed interstitial inflammatory changes in the lungs. Indirect immunofluorescence was used to detect the expression of antinuclear antibody (ANA). Based on the above clinical evidence, we considered her symptoms consistent with mixed CTD. We treated her with prednisone (10 mg per day), hydroxychloroquine (200 mg per day), and mycophenolate mofetil (0.75 g, twice per day).

**Figure 1:**
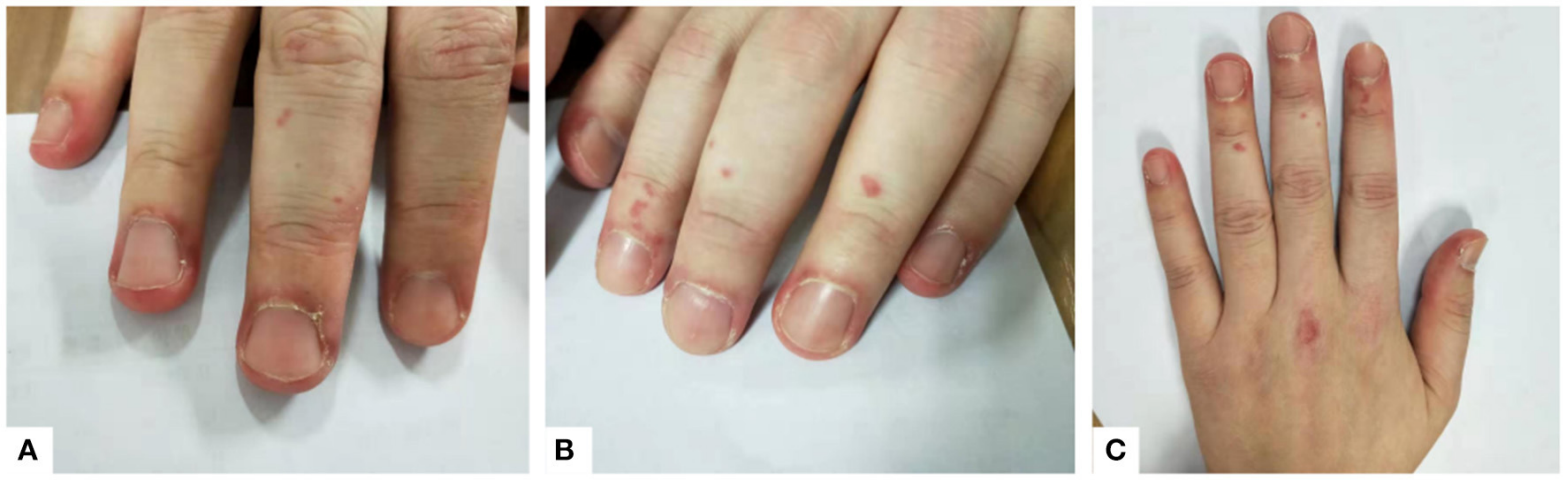
**(A–C)** Erythema around the nail and insignificant Gottron's signs can be seen on the patient's hands.

However, her symptoms were not resolved. On March 2018, the patient was readmitted with aggravated cough and shortness of breath, as well as a rash on both hands, which resembled Gottron's signs (Figure 1). Laboratory test results are detailed in Table 1 (adult reference values in parentheses). Western blot was used to detect the expression of anti-MDA5 antibody. The fluorescence pattern of ANA included nuclear dot (1:3,200), speckled (1:320), and cytoplasmic (1:100). The blood gas analysis was carried out under the condition of oxygen therapy (2 L/min, nasal catheter oxygen inhalation). The critical symptoms and tests of each visit are shown in Table 2. A second pulmonary HRCT scan showed that the pulmonary interstitial inflammatory changes had become more severe, and the patient developed mediastinal and subcutaneous emphysema. At this time, the subcutaneous crepitus was clearly detected in the patient's chest. She had to lie down and receive oxygen therapy (2 L/min, nasal catheter oxygen inhalation) to maintain proper SaO_2_. Once she was active, her SaO_2_ decreased, and her breathing difficulties increased. Her weak physical condition made it impossible for her to carry out her daily life and work, and she could not complete the pulmonary function test due to her poor condition. According to the ADM and RP-ILD diagnostic criteria, she was diagnosed with anti-MDA5 antibody-positive ADM accompanied by RP-ILDs. The patient was prescribed oxygen therapy (changed to 6 L/min, nasal catheter oxygen inhalation to maintain the finger pulse oxygen level over 95%) and methylprednisolone pulse therapy. She was first given high-dose-steroid pulse therapy with methylprednisolone (500 mg per day for 3 days) followed by methylprednisolone (40 mg daily), cyclosporine A (100 mg, twice per day), and hydroxychloroquine (200 mg, twice per day). Since there was no result of bacterial culture, we chose broad-spectrum antibiotics. She was simultaneously prescribed anti-infection treatment with moxifloxacin (400 mg per day for 7 days).

**Table 2:** Main symptoms and laboratory tests results of each stage.

	**Main symptoms**	**Laboratory tests results**
4 months before baseline	Polyarticular pain and swelling in limbs	ANA positive
	Morning stiffness	ESR 43 mm/h
	Fever	
5 months before baseline	Cough	Anti-Ro52 antibody positive
	Shortness of breath	CK and CK-MB normal
	Raynaud's phenomenon	
Baseline	Aggravated cough	Anti-Ro52 antibody positive
	Shortness of breath	Anti-MDA5 antibody positive
	Gottron's signs on both hands	PO2 63.5 mmHg

*ANA, antinuclear antibody; ESR, Erythrocyte sedimentation rate; CK, creatine kinase; CK-MB, Creatine Kinase, MB Form; MDA5, melanoma differentiation-associated gene 5; PO_2_, Partial pressure of oxygen*.

**Figure 2:**
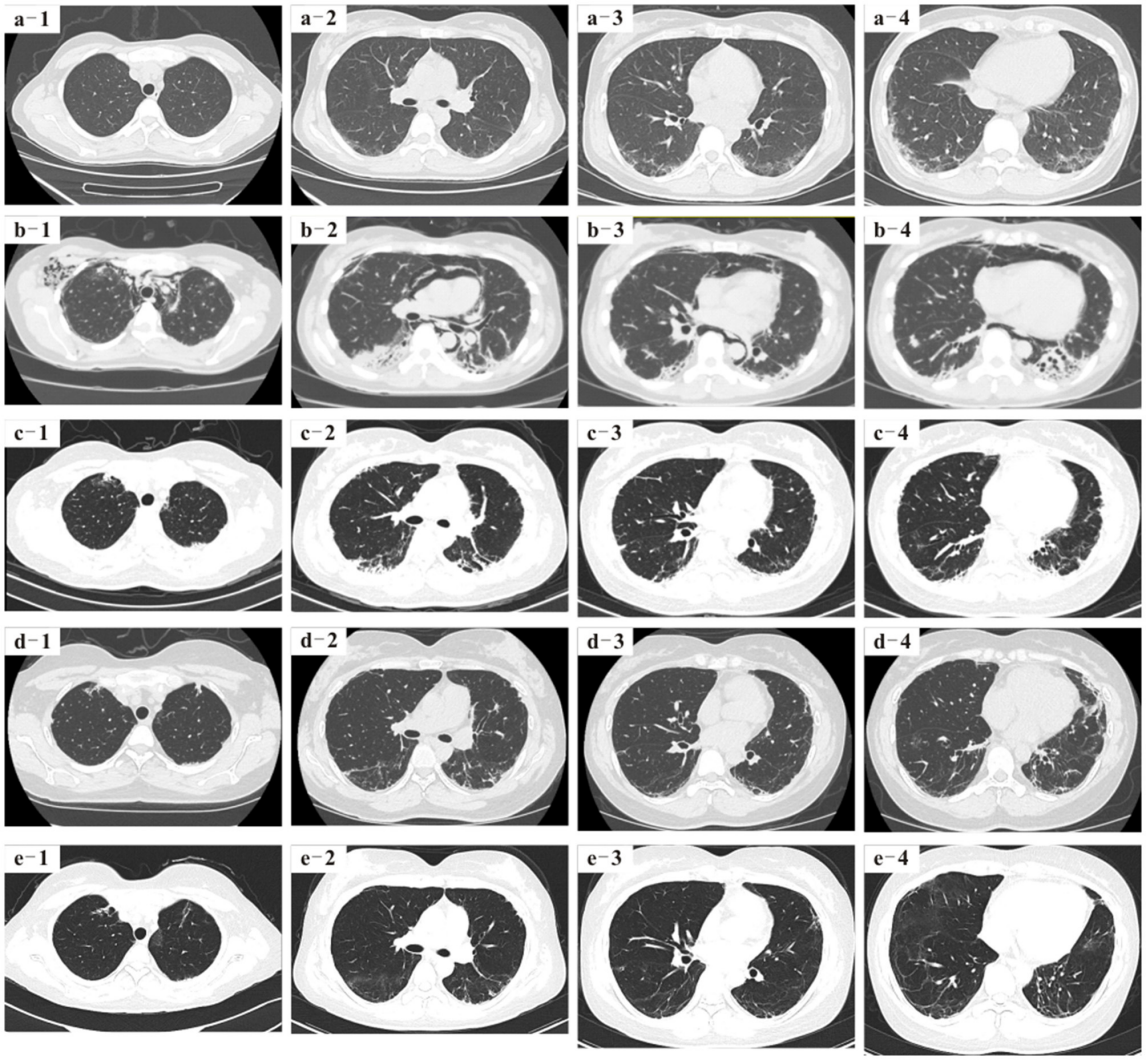
**(a1–a4)** Pulmonary high-resolution CT (HRCT) 4 months before baseline showed interstitial inflammatory changes in the lungs. **(b1–b4)** Pulmonary HRCT of baseline showed that the pulmonary interstitial inflammatory changes were more serious than before, mediastinal and subcutaneous emphysema appeared, and diffuse ground-glass shadows were seen in both lung fields. **(c1–c4)** Pulmonary HRCT reexamination on 4 months after the patient was discharged from our hospital showed that the diffused ground-glass shadows were less than those on March 2018, and mediastinal and subcutaneous emphysema were significantly absorbed. **(d1–d4)** Pulmonary HRCT on 9 months after the patient was discharged from our hospital showed that the lungs were much better than on March 2018. **(e1–e4)** More than 2 years later after the patient was discharged from our hospital, pulmonary HRCT showed lesser ground-glass shadows, and her lungs were much better than before.

Since her discharge from our hospital on March 2018, she has maintained the methylprednisolone therapy (tapered to 10 mg daily), cyclosporine A (100 mg, twice per day), and hydroxychloroquine (200 mg, twice per day). Over the past 2 years, the patient has steadily improved. Pulmonary HRCT scans taken on 4, 9, and 26 months after her discharge from our hospital showed that the interstitial pneumonitis had significantly improved, and mediastinal and subcutaneous emphysema had been gradually absorbed (Figure 2). The patient is a professional translator, and now she can participate in regular work and activities of daily living. Now the patient returns to our hospital regularly for reexamination, and the new pulmonary HRCT scans are being booked.

## Discussion and Conclusion

### Relationship Between Amyopathic Dermatomyositis, Rapidly Progressive Interstitial Lung Diseases, and Anti-melanoma Differentiation-Associated Gene 5 Antibody

A study by Kawasumi et al. on the treatment of IIMs complicated with ILDs could demonstrate that an ILD was a prognostic factor for poor outcomes in DM (17). Huang et al. analyzed the clinical manifestations, serological examination, imaging features, treatment, and prognosis of 32 patients with positive anti-MDA5 antibody, and they concluded that anti-MDA5 antibody was closely related to ILDs and indicated poor prognosis (18). These cases serve as a reminder to treat the pulmonary symptoms of patients with ADM, in addition to the typical dermal and muscular symptoms. The prognosis of patients with ADM and ILD-related complications is poor, so more attention should be paid to the pulmonary changes of clinically ADM (CADM) patients. The question remains as to why ADM often presents with ILD complications. This may be related to the existence of anti-MDA5 antibodies.

Anti-MDA5 antibody is also known as anti-CADM-140 antibody. Sato et al. established an ELISA to detect anti-MDA5 antibody (19). In 2005, Sato et al. found that anti-MDA5 antibody is specific in patients with DM, especially in patients with CADM. In addition, the anti-MDA5 antibody is associated with RP-ILDs (20). A meta-analysis in 2013 showed that compared with anti-MDA5-negative patients, anti-MDA5-positive patients performed a higher prevalence of RP-ILDs (*P* = 0.001) (21). Another meta-analysis released in 2018 showed that the anti-MDA5 antibody was strongly associated with ADM and RP-ILDs (22). The anti-MDA5 antibody was linked to Gottron's sign and papules, mechanic's hand, V rash, skin ulcers, panniculitis, alopecia, arthritis/arthralgia, and pneumomediastinum and accompanied with low risk of muscle weakness, classic DM, and elevated CK (22). Sato et al. found that with the improvement of respiratory symptoms, the titer of anti-MDA5 antibody could be decreased below the critical value (23). Some reported cases have also shown that the titer of anti-MDA5 antibody is closely related to the course of RP-ILDs (20, 24). These suggest that quantitative detection of anti-MDA5 antibody may be helpful to monitor the disease activity of ADM patients with RP-ILDs (23).

### Treatment of Anti-melanoma Differentiation-Associated Gene 5 Antibody-Positive Amyopathic Dermatomyositis Accompanied by Rapidly Progressive Interstitial Lung Diseases

The appropriate management of ILDs is essential to improving the prognosis of patients with DM (17). The treatment of patients who have ADM complicated by RP-ILDs is difficult, and there have been few effective treatment schemes reported. At present, most of the clinical studies on this disease are case reports. ILD is generally treated via either pharmacological or non-pharmacological methods. Common pharmacological treatments include glucocorticoids, immunosuppressants, anti-fibrosis drugs, and cysteine prodrugs, while non-pharmacological treatments consist of oxygen therapy, mechanical ventilation, and plasma exchange (PE). If pulmonary interstitial fibrosis occurs, lung transplantation is currently the most effective treatment. As for MDA, corticosteroids are the only pharmaceutical agents approved by the US Food and Drug Administration for treating myositis (1).

Sato et al. reported a case of RP-ILDs accompanied by anti-MDA-positive ADM. At first, they used the prednisolone pulse therapy (1 g per day for 3 days), and then the prednisolone was maintained at 50 mg daily, and cyclosporine A (100 mg per day) was added to relieve the symptoms of RP-ILDs. After the improvement of clinical symptoms, low-dose prednisolone and cyclosporine A treatment was maintained; after that, no recurrence has occurred for 5 years. Their case supported the view that prednisolone combined with cyclosporine A is effective in the treatment of ADM with RP-ILDs and that using this treatment immediately before respiratory failure occurs can quickly reduce pulmonary symptoms (23). Hamada Ode et al. reported a case of anti-MDA5 antibody-positive ADM accompanied by RP-ILDs in 2015. After five courses of combination therapy with prednisolone, cyclosporine A, and intravenous cyclophosphamide (IVCY), the IVCY treatment was exchanged for high-dose intravenous immunoglobulin therapy (IVIg). Treatment with IVIg improved the symptoms of RP-ILDs and normalized anti-ADM antibody levels, suggesting that IVIg is a promising candidate for the treatment of anti-MDA5 antibody-positive ADM accompanied by RP-ILDs (25). A study published in 2014 by Zou et al. found that basiliximab may improve the survival rate of RP-ILDs in patients with CADM accompanied by anti-MDA5 antibody (8). In 2018, Alqatari et al. reported a case report of MDA-5-associated RP-ILD recurrent pneumothorax. They treated the patient with intravenous steroids, rituximab, tacrolimus, intravenous immunoglobulins, and cyclophosphamide. Unfortunately, the patient died as a result of the rapid progression of the MDA-5-associated RP-ILD (16). In 2020, Abe et al. reported successful treatment of refractory ILDs with anti-MDA5 antibody-positive treated by PE therapy, although the mechanism of PE in the treatment of RP-ILDs is unclear (26).

## Conclusion

Accurate diagnosis is the key to curative effect. At the initial visit, the patient did not exhibit any characteristic symptoms of anti-MDA5 antibody-positive ADM except polyarticular pain in her limbs. Although more Asian patients were reported to have as anti-MDA5 antibody-positive ADM accompanied with RP-ILDs (13, 21), we and the clinicians who treated the patient before did not realize that she might have this disease at that time, since the prevalence of this disease was rarely low. After the following immunological tests, she was diagnosed with CTD. Even the subsequent pulmonary interstitial inflammation was also thought to be a general type of ILD caused by CTD. At present, the treatment of CTD-ILDs is mainly a combination of immunosuppressive drugs, and these treatments depend on clinicians, which vary widely (27). With the Gottron's signs manifesting and the positive anti-MDA5 antibody, she was finally diagnosed as having anti-MDA5 antibody-positive ADM accompanied by RP-ILDs. It was a challenge to diagnose this disease because of its low prevalence.

According to the case reports in recent years, when ADM patients develop RP-ILDs, high-dose methylprednisolone therapy can control the inflammatory response of the lungs in a shorter time. We believe that the use of methylprednisolone pulse therapy creates conditions for the application of immunosuppressants, such as cyclosporine A and hydroxychloroquine. Although PE therapy is also considered as a treatment for anti-MDA5 antibody-positive ADM accompanied by RP-ILDs (26, 28), unfortunately, we did not prescribe the PE therapy for our patient due to the shortage of plasma resources at that time in our hospital. During the 2-years follow-up, we observed a significant improvement in pulmonary symptoms in the patient who was treated with reduced methylprednisolone therapy combined with cyclosporine A and hydroxychloroquine. We learned about the improvement of the patients' pulmonary symptoms through pulmonary HRCT scans but did not continue to carry out quantitative detection of anti-MDA5 antibody. Her condition is gradually under control, which gives her the ability to take care of herself and to do her job as well as she did when she was not sick in the past.

With the progress of medical research, the treatment of anti-MDA5 antibody-positive ADM is also improving. We believe that the treatment of methylprednisolone pulse therapy combined with cyclosporine A and hydroxychloroquine may be an option for the RP-ILDs accompanied by anti-MDA-positive ADM. After the acute phase, long-term low-dose methylprednisolone combined with cyclosporine A and hydroxychloroquine therapy is helpful in the disease control of patients.

## Data Availability Statement

The original contributions presented in the study are included in the article/supplementary material, further inquiries can be directed to the corresponding author/s.

## Ethics Statement

Ethical review and approval was not required for the study on human participants in accordance with the local legislation and institutional requirements. The patients/participants provided their written informed consent to participate in this study. Written informed consent was obtained from the individual(s) for the publication of any potentially identifiable images or data included in this article.

## Author Contributions

Q-CZ and Z-XC contributed to the data collection and in drafting the manuscript. M-YL contributed to the data collection and revised the manuscript. YC reviewed the data and polished the paper. C-SL contributed to the reviewing, revising, and re-writing work. QX conceived of the study and participated in designing, writing, reviewing, and revising of this manuscript. All authors have read and approved the manuscript.

## Conflict of Interest

The authors declare that the research was conducted in the absence of any commercial or financial relationships that could be construed as a potential conflict of interest.

## Abbreviations

MDA5: melanoma differentiation-associated gene 5
ADM: amyopathic dermatomyositis
RP-ILDs: rapidly progressive interstitial lung diseases
ANA: antinuclear antibody
HRCT: high-resolution CT
IIMs: idiopathic inflammatory myopathies
PM: polymyositis
DM: dermatomyositis
CK: creatine kinase
ILDs: interstitial lung diseases
CTD: connective tissue disease
CADM: clinically amyopathic dermatomyositis
IVCY: intravenous cyclophosphamide
IVIg: intravenous immunoglobulin therapy.
